# Treatment patterns, healthcare resource utilization, and costs following first-line antidepressant treatment in major depressive disorder: a retrospective US claims database analysis

**DOI:** 10.1186/s12888-017-1385-0

**Published:** 2017-06-19

**Authors:** Geneviève Gauthier, Annie Guérin, Maryia Zhdanava, William Jacobson, George Nomikos, Elizabeth Merikle, Clément François, Vanessa Perez

**Affiliations:** 1 Analysis Group, Inc., Montreal, QC Canada; 2 Takeda Development Center Americas, Inc., One Takeda Parkway, Deerfield, IL 60015 USA; 3 Sage Therapeutics, Cambridge, MA USA; 4 Covance Inc., Gaithersburg, MD USA; 5 Lundbeck S.A.S, Deerfield, IL USA

**Keywords:** Major depressive disorder, Antidepressants, Treatment patterns, Economic burden, Healthcare resource utilization, Healthcare costs

## Abstract

**Background:**

Although the symptoms of major depressive disorder (MDD) are often manageable with pharmacotherapy, response to first-line antidepressant treatment is often less than optimal. This study describes long-term treatment patterns in MDD patients in the United States and quantifies the economic burden associated with different treatment patterns following first-line antidepressant therapy.

**Methods:**

MDD patients starting first-line antidepressant monotherapy and having continuous enrollment ≥12 months before and ≥24 months following the index date (i.e., the first documented prescription fill) were selected from the Truven Health Analytics MarketScan (2003–2014) database. Based on the type of first treatment change following initiation, six treatment cohorts were defined a priori (“*persistence*”; “*discontinuation*”; “*switch*”; “*dose escalation*”; “*augmentation*”; and “*combination*”). Treatment patterns through the fourth line of therapy within each cohort, healthcare resource utilization (HCRU), and cost analyses were restricted to patients with adequate treatment duration (defined as ≥42 days) in each line (analysis sub-sample, *N* = 21,088). HCRU and costs were described at the cohort and pattern levels. Treatment cohorts representing <5% of the analysis sub-sample were decided a priori not to be analyzed due to limited sample size.

**Results:**

39,557 patients were included. Mean age was 42.1 years, 61.1% of patients were female, and mean follow-up was 4.1 years. Among the analysis sub-sample, the *discontinuation* (49.1%), *dose escalation* (37.4%), and *switch* (6.6%) cohorts were the most common of all treatment cohorts. First-line antidepressant discontinuation without subsequent MDD pharmacotherapy (22.9%) and cycling between discontinuation and resumption (11.2%) were the two most common treatment patterns. Median time to discontinuation was 23 weeks. The *switch* cohort exhibited the highest HCRU (18.9 days with medical visits per-patient-per-year) and greatest healthcare costs ($11,107 per-patient-per-year) following the index date. Treatment patterns representing a cycling on and off treatment in the *switch* cohort were associated with the greatest healthcare costs overall.

**Conclusion:**

A high proportion of patients discontinue first-line antidepressant shortly after initiation. Patterns representing a cycling on and off treatment in the *switch* cohort were associated with the highest healthcare costs. These findings underscore challenges in effectively treating patients with MDD and a need for personalized patient management.

## Background

Major depressive disorder (MDD) is a complex, multifaceted psychiatric condition characterized by a variety of symptoms, including a persistent state of sadness and hopelessness, anhedonia, sleep disturbance, indecision, reduced ability to concentrate, and recurrent suicidal ideation [1, 2]. MDD is one of the most prevalent mental disorders in the United States (US) [3] and a leading cause of global disability [4]. According to the 2014 National Survey on Drug Use and Health, 6.6% of all adults, or 15.7 million people, in the US experienced at least one major depressive episode in the prior 12-month period [5]. The high prevalence and significant functional impact of MDD [6–9] result in considerable economic consequences at all levels of society. Recent evidence shows that the total economic burden of MDD in the US has increased notably in the last decade, up to $210 billion dollars in 2010, with workplace and healthcare costs contributing almost equally (48% and 47%, respectively) followed by suicide-related costs (5%) [10].

Symptoms of MDD are often managed by appropriate psychological and/or pharmacological therapy and should be addressed throughout the depression life-course [11]. Practice guidelines recommend that treatments be individually tailored and based on disease severity and history, level of functioning, and comorbid psychiatric and physical conditions [6, 7, 12, 13]. Antidepressant drugs constitute the standard of care for MDD [6, 7, 14], whereby most patients will receive a selective serotonin reuptake inhibitor (SSRI) as first-line pharmacotherapy [15]. However, response to first-line antidepressant treatment is often not optimal [16–19].

Management of MDD following non-optimal response to pharmacotherapy can be summarized by four key treatment steps as presented in current US guidelines: (1) dose optimization to maximize therapeutic benefit, (2) therapy switch (i.e., discontinuation of the current pharmacotherapy in favor of an alternative pharmacotherapy), (3) combination therapy (i.e., adding a second antidepressant drug to the initial one), or (4) augmentation therapy (i.e., augmenting the initial antidepressant drug with a non-antidepressant pharmacotherapy) [11, 14, 18]. Treatment regimens prescribed in the real-world setting do not always conform to practice guidelines or on-label use of treatment for MDD. Therapeutic decisions are influenced by a number of factors including safety and tolerability, adherence, and patient-physician dynamics [20]. Nonetheless, identification of the most suitable treatment for each patient as early as possible remains a key challenge and represents a barrier to the improvement of long-term treatment outcomes in MDD [7, 20].

An assessment of long-term real-world treatment patterns in MDD can provide empirical evidence on patient burden and illustrate the economic impact associated with different pharmacological approaches taken following initiation of first-line therapy. In this study, we describe long-term real-world treatment patterns among MDD patients in the US and quantify healthcare resource utilization (HCRU) and costs associated with different treatment strategies following first-line antidepressant therapy.

## Methods

### Data source

This study used data from the Truven Health Analytics MarketScan® Database (MarketScan) [21] between January 2003 through March 2014. The MarketScan database is a large US private sector health claims database containing enrollment history, pharmacy and medical (professional and institutional) claims of employees and their dependents, as well as Medicare-eligible retirees with employer-provided Medicare supplemental plans. The database covers all census regions of the United States. MarketScan data are de-identified and comply with the Health Insurance Portability and Accountability Act [22]. No institutional review board approval was necessary for the current study.

### Study population and design

Patients were included if they had at least two claims, within a 6-month period, containing a diagnosis for MDD (ICD-9 codes: 296.2×, 296.3×) and initiated a first-line antidepressant as monotherapy on the same day or within 30 days of a claim containing a diagnosis for MDD. The index date was defined as the first documented prescription fill date for an antidepressant over the period covered by the database and was considered a proxy for first-line antidepressant initiation. Patients were required to be ≥18 years of age as of the index date and continuously enrolled in a healthcare plan for ≥12 months before and ≥24 months after the index date. Patients were excluded if claims for a second antidepressant or an atypical antipsychotic were present within 30 days of the index date. In addition, patients with claims containing a diagnosis for any type of schizophrenic disorder (ICD-9 codes 295.xx) before or after the index date were excluded.

**Fig. 1:**
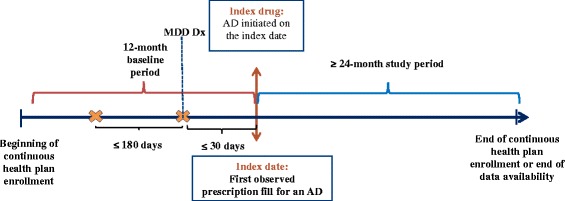
Study design. *MDD = major depressive disorder; Dx = diagnosis; AD = antidepressant

This study was retrospective in design (Fig. 1). The baseline period was defined by the 12-month period before the index date. The study period was defined by the period of continuous healthcare plan enrollment following the index date. A minimum study period of 24 months was required a priori for each patient in order to assess long-term treatment patterns.

### Study measures and treatment outcomes

#### Baseline study characteristics

Sociodemographics, healthcare plan information, the presence of physical and mental comorbidities, all-cause HCRU, number of visits to a mental health professional, and total healthcare costs during the baseline period were summarized among all patients meeting inclusion criteria.

#### Treatment cohorts and patterns

Treatment cohorts were defined based on the type of first treatment change following first-line antidepressant initiation, which included the four key treatment steps for patients with non-optimal response to antidepressant treatment as presented in current US guidelines: dose optimization, switch, combination treatment, and augmentation. For completeness, the present study also examined treatment discontinuation and persistence of initial antidepressant therapy. More specifically, six mutually exclusive cohorts were examined in the present study: (1) *discontinuation* cohort (i.e., patients with an interruption of ≥42 consecutive days of the pharmacotherapy initiated at the beginning of the line of therapy), (2) *dose escalation* cohort (i.e., patients with an increase in daily dose ≥50% compared with the prior dose), (3) *switch* cohort (i.e., patients who initiated a new drug regimen within 42 days of the discontinuation of the prior pharmacotherapy), (4) *persistence* cohort (i.e., patients who continued the first-line antidepressant therapy until the end of the study period), (5) *combination* cohort (i.e., patients who added an antidepressant to the initial antidepressant), and (6) *augmentation* cohort (i.e., patients who added an atypical antipsychotic to initial antidepressant). Definitions for each treatment cohort are presented in Table 1.

**Table 1:** First pharmacotherapy change definition

Pharmacotherapy change^a^	Definition
Discontinuation	Interruption of ≥42 consecutive days of the drug regimen initiated at the beginning of the line of therapy
Dose escalation	Increase in daily dose of ≥50% compared to prior dose - *A dose escalation occurring during the first 21 days was not considered in analyses as this was likely due to titration for tolerability reasons.*
Switch^b^	Initiation of a new drug regimen (antidepressant and/or atypical antipsychotic) within 42 days of the discontinuation of the drug regimen initiated at the beginning of the line of therapy - *Patients on combination therapy at the beginning of a line of therapy who discontinued one treatment but remained on the other treatment were deemed to have switched to a new drug regimen.*
Persistence	Absence of any of the treatment changes until the end of the study period
Combination	Treatment add-on resulting in the use of ≥2 antidepressants simultaneously.
Augmentation	Treatment add-on resulting in the use of one antidepressant and an atypical antipsychotic simultaneously.

^a^Subsequent pharmacotherapy changes (second through fourth) were identified in a similar manner, and also included switch/drop (i.e., in combination and augmentation cohorts only, this represented a change in the overall drug regimen); and resumption of pharmacotherapy (i.e., initiation of a new drug regimen or re-initiation of the same drug regimen following discontinuation)

^b^For antidepressants, the analysis was performed at the active ingredient level, i.e., a change from a branded to a generic form of the same active ingredient was not considered as a switch. A switch from an atypical antipsychotic to another atypical antipsychotic was not considered

Each treatment cohort with the exception of the *persistence* cohort was further evaluated based on observed treatment patterns through the fourth line of therapy. Subsequent treatment changes from second through fourth line of therapy were identified in a similar manner, and, also included ‘switch/drop’ (note, this represented a treatment change in the overall drug regimen in the *combination* and *augmentation* cohorts only) and ‘resumption’ of pharmacotherapy (i.e., initiation of a new drug regimen or re-initiation of the same drug regimen following discontinuation). Each line of therapy was evaluated in terms of adequate treatment duration [11]. In this study, adequate treatment duration was defined as a minimum of 42 days before the occurrence of a treatment change.

The patient sample for analysis was restricted a priori to patients with adequate treatment duration in each line of therapy (analysis sub-sample).

#### Analysis of healthcare resource utilization and costs

Descriptive information on HCRU and costs was reported at the cohort level. Treatment cohorts representing <5% of the analysis sub-sample were not analyzed due to limited sample size, a decision that was made a priori. Within each cohort included in the analysis, HCRU and costs were also examined at the treatment pattern level for the three most common patterns throughout the study period.

The mean and median total number of days with medical visits was estimated during the study period and reported per-patient-per-year (PPPY). The total number of days with medical visits was defined as the sum of inpatient days and days with emergency room visits, outpatient visits, and other medical services (i.e., laboratory, radiology, or other ancillary services). The proportion of the total number of days with mental health-related medical services (i.e., services associated with a claim containing an ICD-9 diagnosis code between 290.xx and 319.xx) was also reported.

The mean and median total healthcare costs incurred during the study period were reported PPPY. The total healthcare cost was defined as the sum of medical costs (combined inpatient, emergency room, outpatient, and other medical service costs) and pharmacy costs (MDD-related pharmacy costs plus other pharmacy costs). The proportion of the total healthcare cost attributable to mental health-related medical services (i.e., medical costs associated with a claim containing an ICD-9 diagnosis code between 290.xx and 319.xx) and MDD-related pharmacy costs (i.e., total pharmacy costs for antidepressants and atypical antipsychotics) were also reported. Healthcare costs were reported in 2014 USD (adjusted for inflation using the Consumer Price Index for medical components) and reflect the total amount reimbursed by insurers and the out-of-pocket costs incurred by patients.

## Results

### Baseline demographic and patient characteristics

**Fig. 2:**
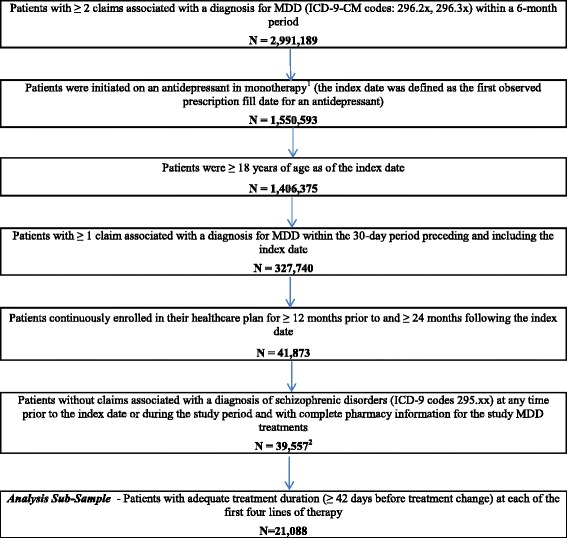
Sample selection flowchart. *MDD = major depressive disorder. **[**1] Patients were considered to be on monotherapy if they did not have any claims for another antidepressant or an atypical antipsychotic during the first 30 days following the index date. [2] Overall study sample without restriction in terms of adequate treatment duration

Inclusion criteria and the number of patients meeting each criterion are presented in Fig. 2. A total of 39,557 MDD patients met all the inclusion criteria and constituted the overall study sample (Fig. 2). The average age was 42.1 years (SD = 15.1) and 61.1% of the overall study sample was female. Most patients were from the western (30.4%) and southern (29.7%) regions of the US and were insured by a preferred provider organization (PPO; 52.4%) (Table 2). Only 2812 patients (7.1%) were covered by a Medicare Supplemental plan at the index date. The prevalence of comorbid conditions was low (average Charlson-Quan Comorbidity Index score = 0.4 ± 1.0). Patients had, on average, a total of two visits to a mental health professional, one outpatient visit per month, and incurred an average annual total healthcare cost of $9941 USD during the baseline period. SSRIs were the most common class of antidepressant drugs initiated on the index date (69.5% of patients). Mean follow-up duration after the index date was 4.1 years (SD = 1.9).

**Table 2:** Sociodemographic and clinical characteristics of patients at baseline

	Overall study sample^a^	Analysis sub-sample
	*N* = 39,557	*N* = 21,088
Age at Index Date, Mean ± SD [Median]	42.1 ± 15.1 [42]	43.3 ± 15.2 [43]
Female, *N* (%)	24,150 (61.1)	12,784 (60.6)
Region of Residence at Index Date, *N* (%)		
West	12,021 (30.4)	6946 (32.9)
South	11,764 (29.7)	5798 (27.5)
North East	10,474 (26.5)	5530 (26.2)
North Central	4773 (12.1)	2572 (12.2)
Unknown	525 (1.3)	242 (1.1)
Type of Healthcare Plan at Index Date^*^, *N* (%)		
PPO	20,747 (52.4)	10,834 (51.4)
HMO and POS with Capitation	10,081 (25.5)	5750 (27.3)
POS without Capitation and EPO	4272 (10.8)	2081 (9.9)
Comprehensive	2605 (6.6)	1438 (6.8)
CDHP and HDHP	1354 (3.4)	740 (3.5)
Unknown	498 (1.3)	245 (1.2)
Medicare Coverage at Index Date, *N* (%)	2812 (7.1)	1723 (8.2)
CCI during the 12-Month Baseline Period, Mean ± SD [Median]^b, c^	0.4 ± 1.0 [0]	0.4 ± 1.0 [0]
Antidepressant Initiated on the Index Date, *N* (%)		
SSRI	27,502 (69.5)	15,048 (71.4)
SNRI	4283 (10.8)	2325 (11.0)
TCA	403 (1.0)	124 (0.6)
Other	7369 (18.6)	3591 (17.0)
All-Cause Healthcare Resource Utilization, Mean ± SD [Median]		
Inpatient Admissions	0.2 ± 0.6 [0]	0.2 ± 0.5 [0]
Outpatient Visits	12.2 ± 12.6 [8]	12.1 ± 12.5 [8]
Emergency Room Visits	0.4 ± 1.0 [0]	0.3 ± 0.9 [0]
Other Medical Service Visits	1.5 ± 3.9 [0]	1.6 ± 4.1 [0]
Number of Visits to a Mental Health Professional, Mean ± SD [Median]	1.9 ± 4.1 [1]	1.8 ± 4.2 [1]
All-Cause Healthcare Costs (2014 USD), Mean ± SD [Median]	9941 ± 48,697 [3375]	9152 ± 23,965 [3230]

*PPO = Preferred Provider Organization; *HMO* = Home Maintenance Organization; *POS* = Point of Service; *EPO* = Exclusive Provider Organization; *CDHP* = Consumer Directed Health Plan; *HDHP* = High Deductible Health Plan; *CCI* = Charlson-Quan Comorbidity Index; *SD* = standard deviation; *SSRI* = Selective serotonin reuptake inhibitor; *SNRI* = Serotonin-norepinephrine reuptake inhibitor; *TCA* = Tricyclic agent

^a^The overall study sample was defined without restriction in terms of adequate treatment duration.

^b^Quan H, Sundararajan V, Halfon P et al. Coding Algorithms for Defining Comorbidities in ICD-9-CM and ICD-10 Administrative Data. Medical Care 2005;43:1130–1139. The CCI score is calculated based on dichotomous variables indicating whether or not a patient had the condition as follows: sum (myocardial infarction, congestive heart failure, peripheral vascular disease, cardiovascular disease, dementia, chronic pulmonary disease, rheumatologic disease, peptic ulcer disease, max[mild liver disease, 3*moderate liver disease], max[mild diabetes, 2*chronic diabetes], 2*hemiplegia, 2* renal disease, max [2* malignancy, 6* metastatic solid tumor], 6*AIDS)

^c^The most common physical comorbidity during the baseline period was hypertension, observed in 7392 (18.7%) patients. The most common mental comorbidity during the baseline period was anxiety disorder, observed in 5698 (14.4%) patients

### Treatment cohorts and patterns

A total of 21,088 patients had at least 42 days of treatment duration for each line of therapy evaluated and constituted the analysis sub-sample (Table 3) – treatment duration of at least 42 days was considered a proxy for adequate treatment duration in this study [11]. The *discontinuation*, *dose escalation*, and *switch* cohorts were the most common treatment cohorts (*discontinuation*, 49.1%; *dose escalation*, 37.4%; and *switch*, 6.6%, respectively). The two most common treatment patterns within the *discontinuation* cohort were discontinuation with no subsequent MDD-related pharmacotherapy (22.9%) and cycling between therapy discontinuation and resumption (11.2%; Table 3) – overall, after discontinuing the initial antidepressant, 16.7% of the patients later resumed one of the studied treatments (Table 3, pattern 1b and 1c). Among patients who discontinued, median time to discontinuation of first-line therapy was 23 weeks. Cycling between therapy discontinuation and resumption following initial dose escalation was also one of the most common treatment patterns in the *dose escalation* cohort. Within the *switch* cohort, the most common treatment pattern was a switch from initial therapy followed by therapy discontinuation without further MDD-related pharmacotherapy (Table 3).

**Table 3:** Treatment cohorts and most common treatment patterns within each cohort

	*N* (%)^a^
Total Number of Patients – Analysis Sub-Sample	21,088 (100%)
Number of patients with 3 most common treatment patterns within each cohort	14,882 (70.6%)
Discontinuation (Cohort 1)	10,358 (49.1)
Pattern 1a: Discontinuation → Remained Untreated	4833 (22.9)
Pattern 1b: Discontinuation → Resumption → Discontinuation → Resumption	2364 (11.2)
Pattern 1c: Discontinuation → Resumption → Discontinuation → Remained Untreated	1158 (5.5)
other patterns	2003 (9.5)
Dose Escalation (Cohort 2)	7879 (37.4)
Pattern 2a: Escalation → Discontinuation → Remained Untreated	1918 (9.1)
Pattern 2b: Escalation → Discontinuation → Resumption → Discontinuation	1866 (8.8)
Pattern 2c: Escalation → Persistence	1104 (5.2)
other patterns	2991 (14.2)
Switch (Cohort 3)	1390 (6.6)
Pattern 3a: Switch → Discontinuation → Remained Untreated	275 (1.3)
Pattern 3b: Switch → Discontinuation → Resumption → Discontinuation	198 (0.9)
Pattern 3c: Switch → Escalation → Discontinuation → Resumption	185 (0.9)
other patterns	732 (3.5)
Persistence (Cohort 4)	732 (3.5)^b,c^
Combination (Cohort 5)	588 (2.8)^c^
Pattern 5a: Combination → Switch/Drop → Discontinuation → Resumption	82 (0.4)
Pattern 5b: Combination → Escalation → Switch/Drop → Combination	58 (0.3)
Pattern 5c: Combination → Switch/Drop → Combination → Switch/Drop	60 (0.3)
other patterns	388 (1.8)
Augmentation (Cohort 6)	141 (0.7)^c^
Pattern 6a: Augmentation → Switch/Drop → Augmentation → Switch/Drop	19 (0.1)
Pattern 6b: Augmentation → Switch/Drop → Discontinuation → Resumption	18 (0.1)
Pattern 6c: Augmentation → Persistence	12 (0.1)
other patterns	83 (0.4)

^a^All percentages were computed among the total number of patients in the analysis sub-sample (*N* = 21,088)

^b^The mean and median duration of treatment for the *persistence* cohort was 37.9 and 33.6 months, respectively

^c^Treatment cohorts representing <5% of the analysis sub-sample were not further analyzed due to limited sample size (decided a priori)

### Healthcare resource utilization and costs

#### Treatment cohort-level

**Fig. 3:**
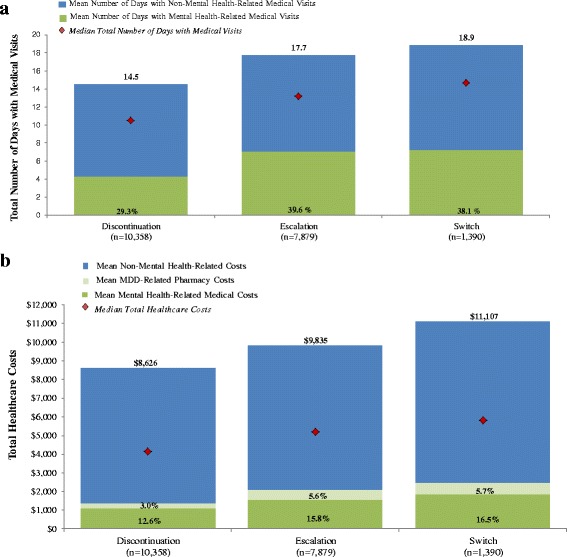
**a** Healthcare resource utilization PPPY during the study period - treatment cohort-level [1]. Data on HCRU are presented for the cohorts that each comprised more than 5% of the analysis sub-sample (decided a priori) [2]. A total number of 14.5 days with medical visits per year in the discontinuation cohort indicates that there was an average of one visit per month, approximately (this interpretation applies across cohorts) [3]. Mean follow-up was 49.6 months in the discontinuation cohort, 49.1 months in the dose escalation cohort, and 50.6 months in the switch cohort [4]. Percentage within each bar represents proportion of days with mental health-related medical visits. **b** Total healthcare costs PPPY during the study period - treatment cohort-level [1]. Data on costs are presented for the cohorts that each comprised more than 5% of the analysis sub-sample (decided a priori) [2]. Mean follow-up was 49.6 months in the discontinuation cohort, 49.1 months in the dose escalation cohort, and 50.6 months in the switch cohort [3]. Percentages within each bar represent proportions of mental health-related medical costs and MDD-related pharmacy costs

HCRU PPPY and costs PPPY for the *discontinuation*, *dose escalation*, and *switch* cohorts, which each comprised more than 5% of the analysis sub-sample, are presented in Fig. 3a and b, respectively. During the study period, the *switch* cohort exhibited the highest level of HCRU on average (18.9 days with medical visits PPPY; Fig. 3a). Less than half (38.1%) of these visits were mental health-related. Similarly, the *switch* cohort incurred the greatest amount of total healthcare costs, on average, during the study period ($11,107 PPPY; Fig. 3b), and less than a quarter (16.5%) of the total healthcare costs were mental health-related.

#### Treatment pattern-level

**Fig. 4:**
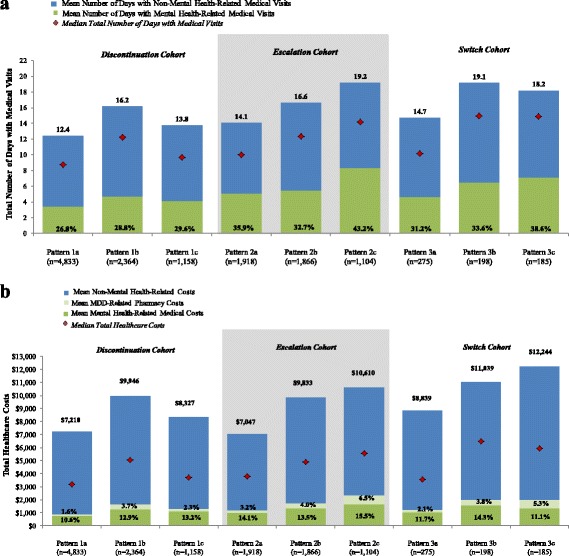
**a** Healthcare resource utilization PPPY during the study period - treatment pattern-level*.* Pattern 1a: Discontinuation→Remained Untreated Pattern 1b: Discontinuation→Resumption→Discontinuation→Resumption Pattern 1c: Discontinuation→Resumption→Discontinuation→Remained Untreated Pattern 2a: Escalation→Discontinuation→Remained Untreated Pattern 2b: Escalation→Discontinuation→Resumption→Discontinuation Pattern 2c: Escalation→Persistence Pattern 3a: Switch→Discontinuation→Remained Untreated Pattern 3b: Switch→Discontinuation→Resumption→Discontinuation Pattern 3c: Switch→Escalation→Discontinuation→Resumption.[1] Data on HCRU are presented only for the three most prevalent patterns within each cohort that comprised more than 5% of the analysis sub-sample (decided a priori). [2] Percentage within each bar represents proportion of days with mental health-related medical visits. **b** Total healthcare costs PPPY during the study period - treatment pattern-level. Pattern 1a: Discontinuation→Remained Untreated Pattern 1b: Discontinuation→Resumption→Discontinuation→Resumption Pattern 1c: Discontinuation→Resumption→Discontinuation→Remained Untreated Pattern 2a: Escalation→Discontinuation→Remained Untreated Pattern 2b: Escalation→Discontinuation→Resumption→Discontinuation Pattern 2c: Escalation→Persistence Pattern 3a: Switch→Discontinuation→Remained Untreated Pattern 3b: Switch→Discontinuation→Resumption→Discontinuation Pattern 3c: Switch→Escalation→Discontinuation→Resumption [1] Data on costs are presented only for the three most prevalent patterns within each cohort that comprised more than 5% of the analysis sub-sample (decided a priori) [2]. Percentages within each bar represent proportions of mental health-related medical costs and MDD-related pharmacy costs

On average, HCRU was the highest among patients who escalated the dose of their first-line therapy and remained on the antidepressant until the end of the study period (pattern 2c in the *dose escalation* cohort: 19.2 days with medical visits, on average, PPPY; Fig. 4a). This estimate was similar to the average number of days with medical visits among patients who switched from their index antidepressant and then cycled on and off treatment (pattern 3b in the *switch* cohort: 19.1 days with medical visits, on average, PPPY; Fig. 4a). Mental health-related medical services accounted for less than half of the overall HCRU in these two treatment patterns (Fig. 4a).

Among the *discontinuation*, *dose escalation*, and *switch* cohorts, average total healthcare costs were highest for patients in the *switch* cohort who switched from their index antidepressant and later cycled on and off treatment during the study period ($12,244 PPPY in pattern 3c followed by $11,039 PPPY in pattern 3b in the *switch* cohort; Fig. 4b). MDD-related pharmacy costs and mental health-related medical costs contributed minimally to the overall cost burden.

## Discussion

This retrospective study provides the first comprehensive characterization of real-world, long-term treatment patterns following initiation of first-line antidepressant therapy among patients with MDD using data from a large US administrative database. This work further represents a novel contribution to the scientific literature by reporting on the economic burden associated with recommended treatment patterns commonly observed in clinical practice in the US.

Our study included a total of 39,557 patients with MDD who were observed for an average of 4.1 years after initiation of first-line antidepressant therapy. Half of the patients had adequate treatment duration in each line of therapy before the occurrence of a treatment change and were included in the analysis sub-sample. Following initiation of first-line therapy, only 3.5% of patients persisted on their initial antidepressant throughout the study period. The most prevalent treatment change following initiation of first-line therapy was treatment discontinuation, which occurred in approximately half (49.1%) of the analysis sub-sample (median time to discontinuation was 23 weeks). Further analysis within this cohort revealed that most patients discontinued their index antidepressant and remained untreated or cycled on and off treatment throughout the study period. Among patients who continued treatment, dose escalation (37.4% of patients) and treatment switch (6.6% of patients) were the most common treatment changes following initiation of first-line therapy. Additionally, our study showed that treatment patterns representing a cycling on and off treatment in the *switch* cohort were associated with the highest mean total healthcare costs. It should be noted that the rate of switch reported in the current study reflects the first treatment change only; accordingly, the overall rate of switch and associated costs throughout the life cycle of treatment is presumably higher.

The study findings show that, in real-world practice, despite the fact that US guidelines recommend treatments to be administered for four to eight weeks before considering a patient as responsive or unresponsive to treatment, a sizable number of patients experience multiple treatment changes after a treatment duration that may not be sufficient to adequately evaluate treatment response [11, 23]. Hence, this study further underscores the need for improved long-term, personalized psychiatric management of patients aimed at yielding the best treatment outcomes possible.

Although most practice guidelines state that pharmacological therapy of patients with MDD typically includes three phases – *acute phase* (6–8 weeks), *continuation phase* (16–20 weeks), and *maintenance phase* for chronic and/or recurrent MDD [23] – our results show that, in real-world practice, a considerable proportion of patients do not reach the end of the *continuation phase* due to treatment discontinuation. Consequently, a significant number of patients could benefit from continuing therapy over a longer period of time assuming no major safety and tolerability issues are of concern.

The high rate of discontinuation in our study is consistent with the high discontinuation rate reported in a recent study by Jung et al. [24]. In this study, over 70% of patients discontinued antidepressants after 6 months (including patients who switched). While some patients may discontinue therapy due to having achieved remission, treatment side effects, inadequate patient follow-up, patient concern about medications and fear of addiction, physician-patient miscommunication, misperception of the benefits of treatment, or patient perception that treatment is no longer needed are also common reasons for discontinuation [25–31]. Indeed, as response to first-line therapy is often less than optimal [16–19], it is likely that the *discontinuation* cohort in our study includes non-responders and non-remitters. Furthermore, some of the symptoms that characterize MDD – such as difficulty planning, disorganization, distraction, and other cognitive impairments – are also likely to contribute to poor persistence on first-line antidepressant and frequent cycling on and off treatment. Although the reasons for therapy discontinuation were not available, and that it is possible that a number of patients discontinued due to successful treatment, the high discontinuation rates and the relatively short period of time within which patients discontinued their first-line antidepressant also suggest that there may be a need to improve long-term treatment persistence in some patients with MDD.

Dose escalation and switching to another antidepressant or to an atypical antipsychotic were found to be the most common treatment changes among patients who continued treatment after first-line therapy. This finding suggests a physician’s preference for antidepressant drug monotherapy versus adding or combining therapies. Although the choice of the next treatment step is likely to depend on the initial treatment response (i.e., partial vs. no response) [11, 32], a physician’s preference for monotherapy in the US could reflect an effort to avoid potential drug interactions and to maximize compliance by minimizing the number of MDD pharmacotherapies prescribed.

In terms of HCRU and costs, our results indicate that treatment patterns representing a cycling on and off treatment in the *switch* cohort were associated with the greatest total healthcare costs PPPY over the long-term follow-up. This may suggest that patients who commonly switch and continually cycle through different treatments might be particularly difficult-to-treat patients, thus driving medical costs up. It should, however, be noted that the treatment patterns observed in this study, along with their outcomes, may not reflect optimal treatment paths and can have grave consequences on patient responses and treatment costs. For example, it may be more appropriate to switch a patient from their first line of treatment rather than escalating their initial dose; this may, for some patients, result in lower burden and reduced long-term costs if treatment selection is appropriately determined early in the treatment path, i.e., it may be more costly and burdensome if a patient remains on a treatment that fails to be effective rather than switching to a different, more optimal, treatment [33]). Results for HCRU and costs also suggest that a relatively high proportion of the medical services and costs are not related to mental health. These results are consistent with prior studies. For example, Simon et al. (1995) reported that 66% to 71% of the total direct healthcare costs were not related to antidepressant or mental health-related visit costs [34]. These findings may reflect HCRU and costs associated with the management of other concurrent comorbidities, complications, or unrelated conditions worth evaluating in future studies. Despite the high direct healthcare costs PPPY reported in this study, these costs likely reflect an underestimate of the overall economic burden reported for MDD in the US. Indeed, a previously published US study estimated that only half of the economic burden of MDD was attributable to direct healthcare costs, with the other half attributable to worker absenteeism and presenteeism [10].

Mention of the methodological limitations of this study is warranted. First, treatment cohorts and patterns were identified based on claims for a filled prescription, which does not guarantee that the medication was consumed by the patient or account for changes in prescribed therapy. Second, a patient’s medical history may influence treatment decisions and subsequent treatment patterns and outcomes. However, disease severity, clinical characteristics and reasons underlying specific treatment patterns are not available in claims databases. Further analyses using a different data source or data linkage with patients’ medical records is warranted to better understand treatment decisions resulting in the patterns observed. Third, the study sample was limited to privately insured employees and their dependents. All patients included in our study sample were required to meet pre-determined inclusion and exclusion criteria (e.g., to have at least two billing claims for MDD within a pre-specified time period and to initiate the index antidepressant in monotherapy as first-line treatment). Accordingly, the results from our study may not be generalizable to the overall MDD patient population in the US. Fourth, our study was descriptive in nature and no statistical comparisons between treatment cohorts/patterns were conducted. However, it is possible that patients across treatment cohorts/patterns have differential disease profiles. Patients may have also undergone concomitant non-pharmacological therapy, such as psychotherapy, which may influence treatment decisions and outcomes. Non-pharmacological treatment like cognitive behavioral therapy was not evaluated as part of the current study. Further analyses are warranted to better understand reasons for specific treatment changes/patterns and to compare outcomes between treatment cohorts/patterns, including cohorts receiving concomitant psychotherapy. Fifth, the current study identifies dose escalation but did not assess whether the dosage prescribed was within the therapeutic range (a determination that would require that the dose prescribed is indeed suitable for a particular patient). Additional analyses would be needed to analyze the impact of adequate/inadequate dosing on outcomes and costs. Sixth, this study was designed to evaluate MDD patients with treatment patterns involving antidepressant and antipsychotic therapies. It is, however, possible that some patients with MDD also receive other treatments such as lithium and thyroid hormones as part of their augmentation therapy. Other therapies such as lithium and thyroid hormones, for example, were not analyzed in our study as patients augmenting with these agents may require them for the treatment of non-MDD conditions (e.g., lithium for bipolar disorder and thyroid hormones for conditions related to hormonal imbalance) – claims data do not include information to confirm the reason for which a treatment is prescribed. Finally, administrative claims databases are subject to coding errors and data omissions.

## Conclusions

This US study provided the first comprehensive characterization of real-world treatment patterns and associated HCRU and costs following initiation of first-line therapy with an antidepressant in patients with MDD. The results showed that an exceptionally small number of patients persisted on their first-line antidepressant throughout the study period. In contrast, nearly half of the analysis sub-sample discontinued therapy following first-line antidepressant, making discontinuation the most common initial treatment change. Discontinuation and resumption of treatment within the *switch* cohort was generally associated with the highest healthcare costs. In conclusion, this study underscores several challenges in effectively treating MDD and an urgent need for personalized patient management aimed at improving quality of care and reducing overall burden.

## Abbreviations

AD: antidepressant
CCI: Charlson-Quan comorbidity index
CDHP: consumer directed health plan
DX: diagnosis
EPO: exclusive provider organization
HCRU: healthcare resource utilization
HDHP: high deductible health plan
HMO: Home Maintenance Organization
MarketScan: Truven Health Analytics MarketScan® Databases
MDD: major depressive disorder
POS: point of service
PPO: preferred provider organization
PPPY: per-patient-per-year
SD: standardized deviation
SNRI: serotonin-norepinephrine reuptake inhibitor
SSRI: selective serotonin reuptake inhibitor
TCA: tricyclic agent
US: United States
USD: United States dollar
